# A Rare Thyroid Metastasis from Uveal Melanoma and Response to Immunotherapy Agents

**DOI:** 10.1155/2016/6564094

**Published:** 2016-03-24

**Authors:** Dearbhaile Catherine Collins, Ruben Yela, Noel Horgan, Derek Gerard Power

**Affiliations:** ^1^Department of Medical Oncology, Cork University Hospital, Wilton, Co. Cork, Ireland; ^2^Department of Histopathology, Cork University Hospital, Wilton, Co. Cork, Ireland; ^3^Department of Ophthalmology, Royal Victoria Eye and Ear Hospital, Dublin 2, Ireland

## Abstract

Thyroid metastasis is a rare occurrence with cutaneous melanoma and even more uncommon with uveal melanoma. The management of such metastasis is uncertain due to its infrequency and, in the era of immunotherapy, the effect of these novel drugs on uncommon metastasis, such as to the thyroid, is unknown. We report the rare case of a thyroid metastasis in a patient diagnosed with ocular melanoma initially managed with enucleation. Metastatic disease developed in the lung and thyroid gland. The case patient received the immunotherapy ipilimumab with stable disease in the thyroid and progressive disease elsewhere. The patient was then further treated with a second immunotherapy agent, pembrolizumab, and remains with stable disease one year later. We discuss the current literature on thyroid metastases from all causes and the optimal known management strategies. Furthermore, we provide an original report on the response of this disease to the novel immunomodulators, ipilimumab, and pembrolizumab with stable disease four years after initial diagnosis of ocular melanoma.

## 1. Introduction

Thyroid metastases are an uncommon occurrence. Reported incidence ranges from 0.5% to 24.2% in autopsy studies [1–5]. They most often arise from renal, breast, and lung primaries; however cutaneous melanoma is also a common site [1, 2, 6]. Diagnosis is most commonly made with ultrasonography and fine needle aspiration cytology (FNAC). Systemic management of the primary disease is the optimal treatment strategy, although thyroidectomy may be necessary for palliative symptom control and, in some malignancies, resection may improve survival [7]. Ocular melanoma comprises 3 to 4% of all malignant melanoma and most commonly metastasizes to the liver [8, 9]. It has only been reported once before in the literature as giving rise to thyroid metastases in 1986 [10]. To add to this scant literature, we report a rare case of thyroid metastasis from uveal melanoma and furthermore report a disease response to the systemic immunotherapies in the form of ipilimumab and pembrolizumab.

## 2. Case Presentation

We present the case of a 63-year-old French national who was residing in Ireland. He was diagnosed with right ocular melanoma and underwent enucleation in a specialist institution in February 2011. Histopathology at this time revealed a superonasal posterior pole uveal melanoma measuring 17 × 12 × 10 millimeters. There was histopathological evidence of extrascleral extension, spindle B cell morphology, and considered stage pT4e. There were no mitoses in 40 high power fields. Tumour involved the overlying retina and 17% of cell revealed monosomy of chromosome 3 using fluorescence in situ hybridisation (FISH) analysis. FISH also indicated normal results for chromosome 8 and MYC abnormalities. At time of initial staging and workup our patient was found to have subcentimetre lung nodules on computed tomography (CT) scanning. These were biopsied and on histopathological assessment were identified as benign on both FNAC and core biopsy. Follow-up scanning with annual CT and positron emission tomography- (PET-) CT revealed stable cold lung nodules and no evidence of metastatic melanoma over a thirty-two-month period.

**Figure 1:**
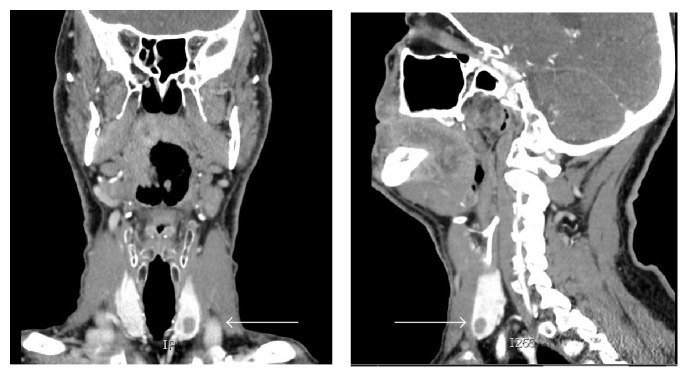
CT neck image of thyroid nodules (identified by white arrows).

In October 2013, he underwent a routine surveillance CT neck, thorax, abdomen, and pelvis, which noted increased size of all lung nodules. For instance, a nodule in the right middle pulmonary lobe measured 8 mm from previous 3 mm. It also identified two new solid lesions in the thyroid, the largest of which was 8 mm in diameter (Figure 1).

**Figure 2:**
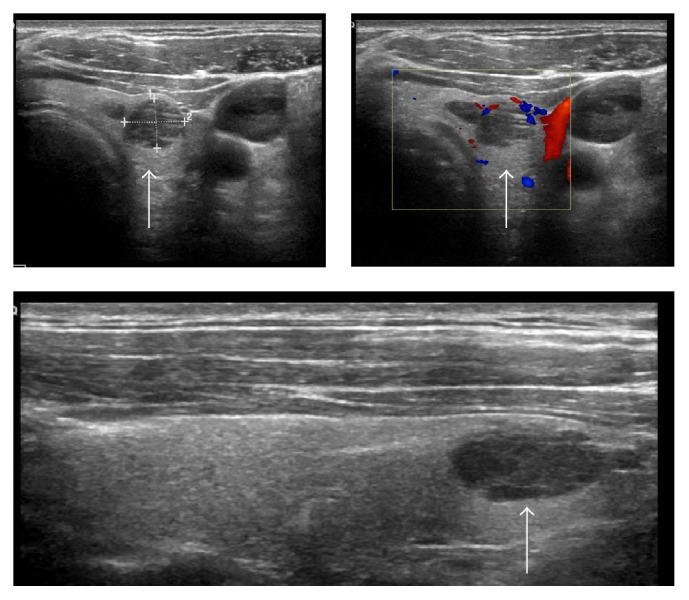
Ultrasound images of thyroid metastasis (identified by white arrows).

Ultrasound guided biopsy of the largest lesion revealed metastatic melanoma (Figure 2).

**Figure 3:**
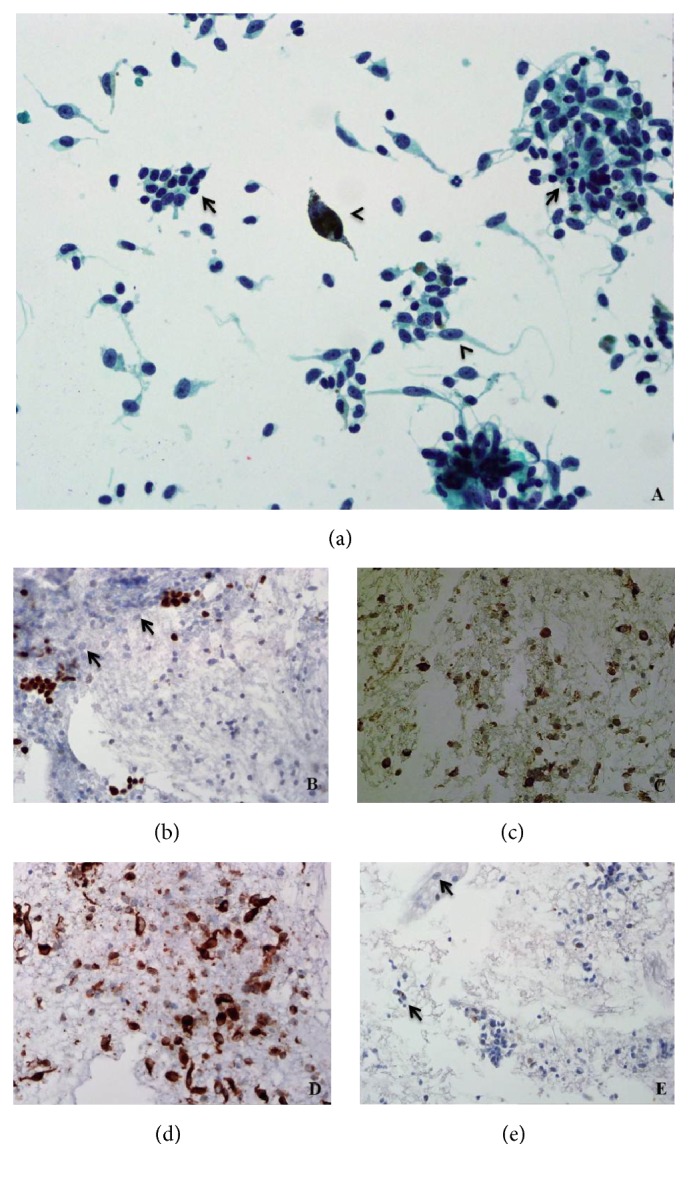
(a) Spindle and epithelioid malignant melanoma cells (arrowheads) admixed with background residual thyroid follicular cells (arrows). Papanicolaou stain, original magnification ×400. (b) TTF-1 highlights residual thyroid follicular cells and is negative in the lesional cells (arrows). TTF-1 ICC, original magnification ×200. (c) HMB45 and (d) MelanA ICC show strong positivity in the lesional cells. Original magnifications ×200. (e) S100 ICC weakly highlights the fine intracytoplasmic pigment present in the lesional cells (arrows). Original magnification ×100.

Pathological examination showed the presence of metastatic spindle and epithelioid malignant melanoma cells (positive staining for HMB45, MelanA, and S100 immunocytochemistry (ICC)) amongst residual thyroid follicles (positive staining for TTF-1 ICC). Some of the metastatic melanoma cells showed fine intracytoplasmic pigment and prominent nucleoli (Figure 3).

**Figure 4:**
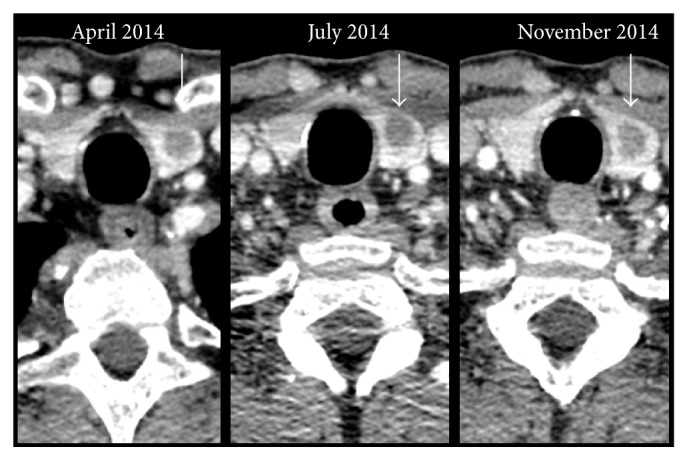
CT neck image of thyroid nodule (identified by white arrows) over time.

The case patient was of protooncogene* braf* mutation wild type and went on to receive four cycles of the immunotherapy, ipilimumab in early 2014, which were tolerated well. Restaging CT one month after the final cycle revealed progressive disease in the lungs, with new disease in the adrenal gland. The thyroid metastases were considered unchanged from prior imaging. The patient was managed with surveillance and further CT scanning three months and six months later revealed stable disease in the thyroid (Figure 4) with new disease infradiaphragmatically. In January 2015, the novel immunomodulator, pembrolizumab, was prescribed at two milligrams per kilogram every three weeks. The patient continues on regular pembrolizumab with all thyroid, pulmonary, and adrenal metastatic disease stable by immune response criteria (iRECIST) at each three-month CT assessment since commencing this therapy.

## 3. Discussion

Thyroid metastases are an uncommon occurrence in clinical practice. Autopsy reports vary considerably between 0.5% and 24% in incidence [1–5]. Primary sources most commonly include carcinomas of the breast, lung, kidney, gastrointestinal tract, head and neck, and malignant melanoma [1, 4, 11]. Renal cell carcinoma [12–14] and breast [15] are the most frequent in the literature. Thyroid metastases usually occur in the presence of metastatic disease elsewhere [7, 12, 14] and, if isolated, are often quickly followed by the development of disseminated disease. Their development can be distant from the primary diagnosis. This is particularly in the case of renal cell carcinoma, where there have been multiple cases of metastasis developing over twenty years after diagnosis of the primary malignancy [11, 16]. The published literature on cutaneous melanoma metastatic to the thyroid gland is limited to case reports or small case series [13, 17–19]. Bozbora et al. reported a case of cutaneous malignant melanoma to thyroid after excision of a two-millimeter melanoma five years before [17]. There is only one cited case report of ocular melanoma metastasising to the thyroid gland [10].

Uveal melanomas differ from cutaneous melanomas in a number of areas. Firstly, ocular melanomas have a predilection to metastasise to the liver along haematogenous drainage pathways although spread can occur to lung, bone, brain, skin, and potentially any site within the body [20]. Factors predictive of metastasis from uveal melanoma include tumour thickness and genetic aberrations. Shields et al. found for each millimeter increase in tumour thickness that there was a resultant increased risk of metastasis of 1.06 [21]. The risk of spread is approximately 6% with uveal melanomas less than or equal to one millimeter in thickness and up to 51% for those greater than ten millimeters. Uveal melanomas also differ from cutaneous lesions in their expression of genetic abnormalities, in particular, anomalies of chromosomes 1, 3, 6, and 8. These abnormalities are prognostic as well as predictive for metastasis [22–24]. As, in our case, monosomy of chromosome 3 is the most frequent chromosomal aberration, reported in about half of patients [25, 26]. Its loss is detected in more than 70% of metastasising and only 20% nonmetastasising uveal melanomas and correlated with reduced patient survival [22–24, 27]. A further difference between uveal melanoma and cutaneous melanoma is that the latter frequently carries oncogenic driver mutations in the proteins Raf and Ras, which results in constitutively activated mitogen associated protein kinase (MAPK) pathway signalling and leads to tumorigenesis, cellular proliferation, and dissemination [28–30]. Although MAPK activation also exists in uveal melanoma it is rarely due to mutations in Raf and Ras [31–34]. Other genetic oncogenic drivers include GNAQ [35–37], GNA11 [38], BAP1 mutations [39], and PTEN (phosphatase and tensin homolog) loss [40]. Whether these mutations actually correlate with overall patient outcome is unconfirmed [36, 41].

Management of thyroid metastasis is varied and depends upon symptoms at presentation. Treatment strategies can include surgical resection or debulking by complete or partial thyroidectomy, radiotherapy, and/or systemic therapy. The aim of surgical intervention should be primarily for compressive symptom control such as dysphagia, dyspnea, and stridor [42]. Thyroid metastasis from other malignancies such as renal cell carcinoma, if resected, may have a cytoreductive role and impact positively upon patient outcome [7]. Metastasectomy in melanoma patients has been shown to confer a significant increase in survival for patients in particular with pulmonary and hepatic metastasis [43–45]. This is irrespective of whether the primary melanoma is cutaneous or uveal [46]. It is unknown whether thyroid metastasectomy, specifically, confers any survival benefit on account of the rarity of this occurrence [7, 12].

In this case report, we describe a rare case of uveal melanoma with metastasis to the thyroid gland. To date, the literature only records one such case previously published in 1986 [10]. We also give an account of the response of this metastatic thyroidal disease to immunotherapy. The malignancy, following four cycles of ipilimumab, remained stable within the thyroid gland although new metastatic deposits developed elsewhere. Ipilimumab is an inhibitor of cytotoxic-T-lymphocyte-associated-antigen 4 (CTLA-4) that results in upregulation of the immune system. It is well established as a treatment for metastatic malignant cutaneous melanoma [47]. One year later, he received pembrolizumab, a novel immunomodulator that targets programmed-death-1 (PD-1) receptor and activates T-cell mediated immune responses against tumour cells. Again pembrolizumab is a proven efficacious treatment in metastatic cutaneous melanoma [48]. He remains well with stable metastatic disease almost one year after commencing this second immunotherapy and four years after his initial diagnosis.

In conclusion, unusual metastasis, for instance, to the thyroid should always be considered in the setting of carcinoma, including uveal melanoma. The disease should be managed according to the primary malignancy with appropriate chemotherapy, biological therapy and radiotherapy, and multidisciplinary discussion. Surgical resection of thyroid metastasis can be considered on a case-by-case basis, keeping in mind the natural biology of the primary malignancy as well as available systemic treatment strategies. Although immunotherapies such as ipilimumab and pembrolizumab are frequently used in cutaneous melanoma, they can be efficacious on metastatic uveal melanoma also.
